# Incidence of a Positive Sentinel Lymph Node Biopsy in Screen-Detected Early Breast Cancer

**DOI:** 10.7759/cureus.74250

**Published:** 2024-11-22

**Authors:** Anu Sandhya, Muhammad Fahim, Aron Kulanathan, Awa Tansie

**Affiliations:** 1 Breast Surgery, Surrey and Sussex Healthcare Trust, Redhill, GBR; 2 General Surgery, Surrey and Sussex Healthcare Trust, Redhill, GBR; 3 General Surgery, Brighton and Sussex Medical School, Brighton, GBR

**Keywords:** axillary node clearance, axillary node dissection, breast cancer, breast conservation surgery, mastectomy, screen-detected breast cancer, sentinel lymph node biopsy

## Abstract

Introduction

Current guidelines advocate for a sentinel lymph node biopsy (SLNB) in patients with invasive breast cancer with negative axillary ultrasonography. However, emerging evidence has contradicted this, and SLNB omission has been found to be non-inferior in selected low-risk breast cancers. This retrospective study aimed to evaluate the incidence of SLNB in screen-detected invasive breast cancer. The secondary outcome was to identify risk factors in patients with positive SLNB and further axillary disease.

Methods

All screen-detected histologically confirmed invasive breast cancer and no evidence of spread to ipsilateral axillary lymph nodes (LNs) on ultrasound scans referred from screening between January 2018 and December 2019 were included in the study. All patients underwent surgical excision of the tumor as either breast conservation surgery or mastectomy, along with sentinel node biopsy. SLNB was performed using the dual technique of radioactive dye and blue dye.

Results

One hundred forty-nine patients were included in the study, all of whom were females. The mean patient age was 61.9 years old, with the majority 65 (43.6%) of the patients being in their 60s. Breast-conserving surgery (BCS) was performed in 138 (92.6%) patients. Eighty (53.7%) patients presented with right breast cancer. The mean size of invasive cancer was 15 mm. The mean total tumor size, including invasive and in situ, was 20.9 mm. One hundred twenty-seven (85.2%) patients had unifocal presentations, 69 (46.3%) of tumor foci were in the upper outer quadrant (UOQ), 122 (81.9%) of all tumors were ductal carcinoma, 81 (54.4%) patients had histologically grade 2 carcinomas, 135 (90.6%) of all patient tumors were ER-positive, HER2-negative, and six (4%) were ER-positive, HER2-positive. Twelve (8.05%) out of 149 included patients had positive sentinel LN biopsy. Of those 12 patients, eight (66.7%) had one to two nodes sampled, three (25%) had three to four nodes, and one (8.3%) had five or more nodes sampled. Out of 12 positive SNB patients, 11 had completed axillary node clearance (ANC) as per the National Institute for Health and Care Excellence (NICE) guidelines: nine (81.8%) had no further disease, and two (18.2%) had four positive nodes. The mean number of nodes taken in ANC was 15.8 ± 11.5. Of the two patients with positive axillary disease, one had BCS, and the other had a mastectomy. Both were grade 3 IDC, and the mean size was 57.5 mm. Nine patients died within four years of diagnosis, with four due to distant breast metastasis.

Conclusion

Only 8% of patients had positive SLNB in screen-detected breast cancer, which may support the previous studies of omitting SLNB being non-inferior but only in selected postmenopausal small early breast cancers with normal axillary ultrasound in the absence of any other risk factors. However, close follow-up will be required for disease-free survival, overall survival, and locoregional recurrence in this cohort.

## Introduction

For over a century, surgical intervention for breast cancer has been based upon the Halstedian theory. Subsequently, it has become evident that the biological characteristics of breast cancer, rather than the magnitude of surgical intervention, serve as a significant risk factor for both systemic and locoregional recurrences, thereby supporting the exploration of de-escalation of the extent of surgical intervention [1,2].

In patients with a normal ultrasound scan, a sentinel lymph node biopsy (SLNB) is utilized as the standard technique for axillary staging [3]. Growing bodies of evidence suggest that a significant proportion of patients with clinically negative yet histologically positive lymph nodes on SLNB will undergo completion axillary lymph node dissection (ALND) with no additional nodal metastases identified. In spite of this, clinical practice remains unchanged, with ALND still considered the gold standard.

There have been discussions regarding the over-treatment of the axilla since the American College of Surgeons Oncology Group Z0011 study in 2010 [4,5]. The findings of this randomized clinical trial indicated that there was no discernible benefit associated with ALND in comparison to the omission of ALND, even in instances where a maximum of two sentinel nodes were positive, for patients undergoing breast-conserving surgical procedures, along with adjuvant radiotherapy and endocrine treatment. These findings were corroborated by additional randomized clinical trials and subsequently formed the foundational basis for the establishment of a revised standard in axillary management [6,7]. The Italian SOUND trial, which was a prospective noninferiority phase 3 randomized clinical trial conducted in Italy, Switzerland, Spain, and Chile, was the earliest to open in 2012 and completed accrual in 2017. The results of this study suggested that the omission of axillary surgery was non-inferior to SLNB in patients with early breast cancer and negative axillary lymph nodes on ultrasonography [8].

In our retrospective descriptive study, our primary aim was to evaluate the incidence of positive sentinel lymph nodes in screen-detected early invasive breast cancer patients with negative axillary ultrasounds who underwent surgical treatment via either breast-conserving surgery (BCS) or mastectomy and SLNB. Our secondary outcome was to identify patient and tumor characteristics in patients with positive sentinel nodes and further nodal disease on ALND, which may serve as potential risk factors.

## Materials and methods

Clinical coding was utilized to identify all patients referred from breast screening units to Surrey and Sussex NHS Trust, Redhill, UK, between January 2018 and December 2019. Patients’ electronic records were reviewed and included in a computerized database if they met the inclusion criteria of having a histologically confirmed invasive breast cancer, negative axillary ultrasound for nodal disease or negative core biopsy of axillary lymph nodes, and if they underwent surgical management. Patients were excluded if they had positive pre-operative ultrasonography for axillary disease or core biopsy-proven axillary disease, did not undergo surgical management, or had undergone a previous axillary clearance.

All patients had ultrasound scans of the axilla and/or biopsy if required, and the axillary lymph node status was documented pre-operatively. Patients underwent multidisciplinary team (MDT) discussion pre-operatively, and standard treatment plans were discussed and devised in line with UK national guidelines, which are set by the National Institute for Health and Care Excellence (NICE) [3]. All patients included in our study underwent surgical excision of the tumor as either breast conservation surgery or mastectomy with SLNB. SLNB was performed using a dual technique of radioactive dye and blue dye, and specimens were sent for histological analysis. All patients then had MDT discussions post-operatively once histological data was available, and further management plans were formulated. ALND was offered to all patients with evidence of at least one macrometastasis on SLNB in accordance with NICE guidelines. All surgeries were performed as day case procedures. The data was collected retrospectively and recorded on a pre-designed proforma. Attributes included within our database included age, gender, side of breast cancer, affected quadrant, focality, surgical approach, invasive tumor size, total tumor size, tumor grade, sentinel nodes sampled, number of macrometastasis, number of micrometastasis, number of isolated tumor cells, number of nodes sampled on axillary clearance (where applicable), and hormone receptor status.

Continuous variables were reported as means with standard deviations and categorical data as frequencies with percentages as appropriate. Univariate statistical analysis was utilized to assess for differences in patient demographics and tumor characteristics between those with positive and negative sentinel lymph node biopsies as well as those with and without further nodal disease illustrated on axillary clearance. Comparison between groups was made using a T-test for continuous variables and a chi-square test for categorical variables. Additionally, subgroup analysis was performed on patients with positive sentinel lymph nodes to assess for differences in demographics and tumor characteristics in patients with and without further disease illustrated on axillary clearance. Data was collected using Microsoft Excel and analyzed using IBM SPSS Statistics for Windows, version 28.0 (IBM Corp., Armonk, NY). For all statistical tests performed, statistical significance was set at p < 0.05.

This study did not require ethical approval by an independent research ethics committee, as this is an observational study with all data collected retrospectively and through standard clinical practice.

This manuscript was prepared in accordance with Strengthening the Reporting of Observational Studies in Epidemiology (STROBE) guidelines.

## Results

One hundred sixty patients were screened for eligibility, with 11 being excluded. Eight were excluded due to having positive axillary disease on ultrasonography, two did not undergo surgical management, and one had previously undergone an axillary clearance.

One hundred forty-nine patients were included in the study, all of whom were female. Twelve (8.05%) patients had positive SLNB, with 11 (7.38%) progressing to completion ALND. The mean patient age was 61.9 years old, with the majority (65, 43.6%) of patients in their 60s. BCS was performed in 138 (92.6%) of all patients and nine (75%) of SLNB-positive patients. In the two clearance-positive cases, one had BCS, and the other had a mastectomy. Table 1 summarizes the patient demographic data of patients included in our study.

**Table 1:** Patient demographics SLNB, sentinel lymph node biopsy; ALND, axillary lymph node dissection

Characteristics	SLNB only (N = 149)	Positive on SLNB (N = 12)	Completion ALND (N = 11)	Positive on ALND (N = 2)
Age (years)				
Mean ± SD	61.9 ± 8.2	58.1 ± 7.4	59.2 ± 7.6	54 ± 8.5
Median (range)	62 (46-85)	58.5 (48-69)	60 (48-69)	54 (54-60)
Distribution (N, %)				
40-49 years	7 (4.7)	2 (16.7)	2 (18.2)	1 (50)
50-59 years	53 (35.6)	4 (33.3)	3 (27.3)	-
60-69 years	65 (43.6)	6 (50)	6 (54.5)	1 (50)
≥70 years	24 (16.1)	0 (0)	0 (0)	-
Type of breast surgery (N, %)				
Breast-conserving surgery	138 (92.6)	9 (75)	8 (72.7)	1 (50)
Mastectomy	11 (7.4)	3 (25)	3 (27.3)	1 (50)

The mean size of invasive cancer was 15 mm, and total excised tumors, including DCIS, LCIS, and other in-situ components, were 20.9 mm. Eighty (53.7%) of patients presented with right breast cancer; however, seven (58.3%) of patients with positive SLNB had left-sided breast cancer. The side incidence of the two positive clearance patients was even between the right and left breast. One hundred twenty-seven (85.2%) of all patients had unifocal presentations, and nine (75%) SLNB-positive patients were unifocal. For multi-focal samples, which involved multiple quadrants, the largest foci were used for quadrant incidence calculations. Of the 149 patients involved in this study, 155 quadrants were affected. Sixty-nine (44.5%) of tumor foci were in the upper outer quadrant (UOQ), 33 (21.3%) central, 19 (12.3%) upper inner quadrant (UIQ), 16 (10.3%) lower outer quadrant (LOQ), 16 (10.3%) lower inner quadrant (LIQ), and two (1.3%) other. A similar incidence pattern was seen in those positive at SLNB, with seven (46.7%) patients in the UOQ. Table 2 summarizes the tumor characteristics of our patient cohort.

**Table 2:** Tumor characteristics SLNB, sentinel lymph node biopsy; ALND, axillary lymph node dissection; UOQ, upper outer quadrant; UIQ, upper inner quadrant; LOQ, lower outer quadrant; LIQ, lower inner quadrant ^[1]^Two individuals had bilateral breast cancer, counted as individual cases ^[2]^Ultrasound report specified lateral and outer ^[3]^The histology size of one patient was given as 80+, with the size taken to be 80 mm

Characteristics	SLNB only (N = 149)	Positive on SLNB (N = 12)	Completion ALND (N = 11)	Positive on ALND (N = 2)
Side of tumor incidence (N, %)				
Left^^[1]^^	69 (46.3)	7 (58.3)	7 (63.6)	1 (50)
Right	80 (53.7)	5 (41.7)	4 (36.4)	1 (50)
Focality of tumor (N, %)				
Unifocal	127 (85.2)	9 (75)	8 (72.7)	2 (100)
Multifocal	22 (14.8)	3 (25)	3 (27.3)	0 (0)
Quadrant of tumor incidence (N, %)				
Central	33 (21.3)	2 (13.3)	2 (14.3)	1 (50)
LOQ	16 (10.3)	1 (6.7)	1 (7.1)	-
LIQ	16 (10.3)	1 (6.7)	1 (7.1)	-
UOQ	69 (44.5)	7 (46.7)	7 (50)	1 (50)
UIQ	19 (12.3)	3 (20)	2 (14.3)	-
Other^^[2]^^	2 (1.3)	1 (6.7)	1 (7.1)	-
Invasive tumor size (mm)^^[3]^^				
Mean	15.0 ± 10.9	17.9 ± 19.4	17.9 ± 20.1	57.5 ± 31.8
Median (range)	13 (0.1-80)	11.8 (2-80)	15 (2-80)	57.5 (35-80)
No data (N, %)	5 (3.4)	1 (8.3%)	1 (9.1)	-
No residual after neo-adjuvant therapy (N, %)	4 (2.7)	-	-	-
Total tumor size (mm)				
Mean	20.9 ± 17.7	22.7 ± 19.8	23.3 ± 20.6	57.5 ± 31.8
Median (range)	15 (2-135)	15.3 (7.2-80)	15 (7.2-80)	57.5 (35-80)
No data (N, %)	14 (9.4)	-	-	-
No residual after neo-adjuvant therapy (N, %)	4 (2.7)	-	-	-

One hundred twenty-two (81.9%) of all tumors were ductal carcinoma only, 16 (10.7%) were lobular carcinoma, and 11 (7.4%) were mixed on post-excision histology. All SLNB-positive were IDCs. Eighty-one (54.4%) of patients had histologically grade 2 carcinomas with a similar incidence in SLNB-positive patients. One hundred thirty-five (90.6%) of all patient tumors were ER-positive, HER2-negative and six (4%) ER-positive, HER2-positive. However, all SLNB-positive patients were ER-positive, HER2-negative. Tumor histology is summarized in Table 3.

**Table 3:** Tumor histology SLNB, sentinel lymph node biopsy; ALND, axillary lymph node dissection; ER, estrogen; HER-2, Herceptin-2

Characteristics	SLNB only (N = 149)	Positive on SLNB (N = 12)	Completion ALND (N = 11)	Positive on ALND (N = 2)
Tumor grade (N, %)				
T1	51 (34.2)	4 (33.3)	3 (27.3)	-
T2	81 (54.4)	6 (50)	6 (54.5)	-
T3	17 (11.4)	2 (16.7)	2 (18.2)	2 (100)
Tumor invasive histologic type (N, %)				
Ductal carcinoma, no special type	122 (81.9)	12 (100.0)	11 (100.0)	2 (100)
Lobular carcinoma	16 (10.7)	-	-	-
Other including mixed	11 (7.4)	-	-	-
Tumor receptor type (N, %)				
ER-positive, HER2-negative	135 (90.6)	12 (100)	11 (100)	2 (100)
ER-positive, HER2-positive	6 (4.0)	-	-	-
ER-negative, HER2-positive	2 (1.3)	-	-	-
ER-negative, HER2-negative	6 (4.0)	-	-	-

Ninety-nine (66.4%) of all patients had one to two nodes sampled, 42 (28.2%) had three to four nodes, and eight (5.4%) had five or more nodes sampled. Similar trends were seen in SLNB-positive patients, with eight (66.7%) having one to two nodes removed. Twelve (8.1%) of all patients had positive macro metastases sentinel nodes. Nine (6.0%) patients had one positive node, two (1.3%) patients had two positive nodes, and one (0.7%) patient had greater than three positive nodes. Out of the 11 patients who had completed axillary node clearance (ANC), nine (81.8%) had no further disease, and two (18.2%) had four positive nodes. The mean number of nodes taken in ANC was 15.8 ± 11.5. Table 4 details sentinel lymph node characteristics amongst our patient cohort.

**Table 4:** Sentinel node characteristics SLNB, sentinel lymph node biopsy; ALND, axillary lymph node dissection; ITC, isolated tumor cell

Characteristics	SLNB only (N = 149)	Positive on SLNB (N = 12)	Completion ALND (N = 11)	Positive on ALND (N = 2)
Sentinel lymph nodes removed (N, %)				
1 or 2	99 (66.4)	8 (66.7)	8 (72.7)	-
3 or 4	42 (28.2)	3 (25)	2 (18.2)	1 (50)
≥5	8 (5.4)	1 (8.3)	1 (9.1)	1 (50)
Mean ± SD	2.4 ± 1.45	2.5 ± 1.68	2.5 ± 1.74	5 ± 2.8
Median (range)	2 (1-9)	2 (1-7)	2 (1-7)	5 (3-7)
Sentinel lymph node macrometastases (N, %)				
0	137 (91.9)	-	-	-
1	9 (6.0)	9 (75)	8 (72.7)	-
2	2 (1.3)	2 (16.7)	2 (18.2)	1 (50)
≥3	1 (0.7)	1 (8.3)	1 (9.1)	1 (50)
Mean ± SD	0.12 ± 0.52	1.5 ± 1.12	1.55 ± 1.16	3.5 ± 2.1
Median (range)	0 (0-5)	1 (1-5)	1 (1-5)	3.5 (2-5)
Sentinel lymph node micrometastases (N, %)				
0	142 (95.3)	11 (91.7)	10 (90.9)	2 (100)
1	7 (4.7)	1 (8.3)	1 (9.1)	-
Sentinel lymph node ITCs (N, %)				
0	146 (98.0)	12 (100)	11 (100)	2 (100)
1	3 (2)	-	-	-

Patients with positive sentinel lymph nodes tended to be younger; however, this was not statistically significant (p = 0.10). Univariate analysis revealed no significant difference in either invasive (p = 0.28) or total tumor size (p = 0.83) between patients with positive and negative sentinel lymph node biopsies. Furthermore, there were no significant differences in tumor grade, breast cancer focality, affected quadrant, or hormone receptor status. Table 5 illustrates patient and tumor demographics in patients with positive and negative SLNB.

**Table 5:** Comparison of patients and tumor characteristics with positive and negative SLNB SLNB, sentinel lymph node biopsy; UOQ, upper outer quadrant; UIQ, upper inner quadrant; LOQ, lower outer quadrant; LIQ, lower inner quadrant

Characteristics	Negative nodes on SLNB (N = 137)	Positive nodes on SLNB (N = 12)	p-value
Age (years)			
Mean ± SD	62.2 ± 8.28	58.1 ± 7.42	0.10
Median (range)	63 (46-85)	58.5 (48-69)	
Affected breast (N, %)			
Left	62 (45.3)	7 (58.3)	0.38
Right	75 (54.7)	5 (41.7)	
Tumor extent (mm)			
Invasive ± SD	14.7 ± 9.73	17.9 ± 19.4	0.28
Total ± SD	20.9 ± 17.7	22.7 ± 19.8	0.83
Tumor grade (N, %)			
1	47 (34.3)	4 (33.3)	0.83
2	75 (54.7)	6 (50)	
3	15 (10.9)	2 (16.7)	
Focality (N, %)			
Unifocal	118 (86.1)	9 (75)	0.30
Multifocal	19 (13.9)	3 (25)	
Quadrant (N, %)			
Central	31 (22.1)	2 (13.3)	0.36
LIQ	15 (10.7)	1 (6.7)	
LOQ	15 (10.7)	1 (6.7)	
UIQ	16 (11.4)	3 (20)	
UOQ	62 (44.3)	7 (46.7)	
Other	1 (0.7)	1 (6.7)	
Estrogen receptor status (N, %)			
Positive	129 (94.2)	12 (100)	0.39
Negative	8 (5.8)	0 (0)	
Herceptin receptor status (N, %)			
Positive	8 (5.8)	0 (0)	0.39
Negative	129 (94.2)	12 (100)	

The two patients with further positive nodes on axillary clearance had significantly larger invasive (p < 0.001) and total tumor (p < 0.001) sizes, with more aggressive grades of tumor (p < 0.01) compared to those with no further disease illustrated on axillary clearance (Table 6).

**Table 6:** Comparison of patient and tumor characteristics in patients with and without further nodal metastases noted on ALND ALND, axillary lymph node dissection **p < 0.01, ***p < 0.001

Characteristics	Negative nodes on ALND (N = 9)	Positive nodes on ALND (N = 2)	p-value
Age (years)			
Mean ± SD	59.6 ± 7.54	54 ± 8.49	0.38
Median (range)	60 (49-69)	54 (48-60)	
Affected breast (N, %)			
Left	6 (66.7)	1 (50)	0.66
Right	3 (33.3)	1 (50)	
Tumor extent (mm)			
Invasive	11.8 ± 7.15	57.5 ± 31.8	<0.001***
Total	15.5 ± 6.09	57.5 ± 31.8	<0.001***
Tumor grade (N, %)			
1	4 (40)	0 (0)	0.002**
2	6 (60)	0 (0)	
3	0 (0)	2 (100)	

Four patients died within four years due to metastatic disease. On further analysis, these four patients all had T2 and T3 disease, which was understated on screening imaging. Two were HER2-positive, and two had grade 3 disease. All four of these patients had negative sentinel lymph node biopsies.

## Discussion

Our study found that in this selected cohort of histologically confirmed patients with negative axillary ultrasonography, the yield of SLNB was low, with only 8% of patients having evidence of one or more positive sentinel nodes. SLNB is recommended within the UK by NICE in patients with screen-detected histologically confirmed invasive breast cancer with negative axillary ultrasonography [3]. This low yield on SLNB has been investigated by the SOUND trial, a multi-center noninferiority randomized control trial, which found that in a select cohort of patients with early breast cancer (<20 mm) and negative axillary ultrasonography, an observational approach was non-inferior as opposed to undertaking axillary management in the form of SLNB [8]. Our study also found a low yield of positive sentinel lymph nodes (8% in our study compared with 13.7% in the SOUND trial). However, our cohort of patients incorporated patients with larger mean tumor sizes (>20 mm) and those with multifocal disease, which were exclusion criteria as part of the SOUND trial. Additionally, our study only incorporated a small cohort of patients at a single institution, while the SOUND trial was a large-scale multi-center trial with almost 10 times as many patients included. A key sentiment of the SOUND trial was that the omission of SLNB may be considered in select cases where further clinicopathological data was unlikely to affect post-operative treatment options. As such, further high-quality studies exploring long-term follow-up for disease-free survival, overall survival, and locoregional recurrence are required in the unselected cohort to ascertain whether the omission of sentinel node biopsy is also non-inferior in all patients with negative axillary ultrasonography. 

Additional guidance from NICE advocates for the treatment of axillary disease via either axillary clearance or radiotherapy in patients with evidence of at least one macrometastasis on SLNB [3]. The omission of further axillary surgery in cases of micrometastasis in sentinel nodes has already been established and is incorporated as part of existing guidelines [3,8-10]. However, the notion that sentinel lymph nodes positive for macrometastasis require further treatment was first challenged by the Z0011 trial in 2010, which found that in patients with T1 or T2, N0, and M0 disease, eliminating further axillary management from the treatment algorithm did not result in statistically significant rates of local or regional recurrence [4]. The findings of this study have since been corroborated by other randomized control trials, which have echoed the same sentiment [11-13]. Our study found that in patients with screen-detected histologically confirmed breast cancer, the majority of patients had no evidence of axillary disease, with only 8% of patients having positive SLNB. In all, 16.7% of patients with positive SLNB had evidence of further nodal disease on ALND. While, on the face of it, this may seem to go against the findings of the Z0011 trial, it should be noted that within our study, the two participants with evidence of further metastatic disease on axillary clearance had T3 disease. As such, these patients would not have been eligible candidates within the Z0011 trial. When looking at a comparative cohort to the Z0011 trial (i.e., T1 or T2, N0, and M0 disease), our rate of further axillary disease was 0%, which reaffirms the opinion that in a selected cohort of patients, axillary clearance is an additional and unnecessary procedure with limited prognostic benefit.

Another finding of our study was that patients with further axillary disease beyond SLNB tended to have larger invasive and total tumor sizes as well as higher tumor grades. Our study found that patients with nodal disease tended to have invasive tumor sizes greater than 26 mm and had grade 3 tumors. Meretoja et al. established that in the ultrasonography negative cohort, histological size, multifocality, lymphovascular size, and palpability of tumor were potential risk factors for further axillary disease [14]. The findings of our study corroborate the findings of Meretoja et al. and add in the possibility of higher tumor grade as another potential risk factor for axillary disease, which was also found by Xiong et al. [15]. However, it should be noted that our study only included two patients with further axillary disease, and as such, it was limited by a small sample size.

The majority of patients included in our study were aged between 50 and 70 years of age, which reflects the national screening mammogram program offered within the UK. Another finding from this study is the prevalence of screen-detected breast cancer in the upper outer quadrant of the breast and its correlation with the possibility of the involvement of axillary lymph nodes. In our study, it was noted that 46.7% of patients with positive nodal disease on SLNB had their primary tumor in the upper outer quadrant, which has been described in the literature as a risk factor for axillary lymph node metastases [15].

The main limitations of this study are the design of the study and the fact that data was collected retrospectively from hospital records. Additionally, this study was conducted at a single center with a small sample size, and as such, it is not possible to say whether these findings are applicable to the wider population. Finally, this study did not include disease-free survival, locoregional recurrences, overall survival, and outcomes related to different non-surgical treatments, which can significantly affect outcomes.

## Conclusions

In conclusion, this study proposes that the yield of sentinel node biopsy and further axillary clearance in patients with screen-detected early breast cancer is very low, and omission of sentinel node biopsy could be considered in low-risk T1, postmenopausal, small tumors, which are hormone positive, HER2-negative, provided patients are compliant with the adjuvant treatment of radiotherapy and/or endocrine treatment. However, close follow-up will be required for disease-free survival, overall survival, and locoregional recurrence in this cohort.

Additionally, this study reaffirms that in patients with lower-grade tumors with smaller breast cancers, even in the setting of positive sentinel biopsy, axillary clearance can also be avoided. Higher grade tumors, larger invasive and total tumor sizes, were significantly more likely to result in further nodal disease, and these characteristics may serve as potential risk factors; however, further high-quality trials are required to corroborate these findings.
